# Effect of the Entorhinal Cortex on Ictal Discharges in Low-Mg^**2+**^-Induced Epileptic Hippocampal Slice Models

**DOI:** 10.1155/2014/205912

**Published:** 2014-03-03

**Authors:** Ye-Jun Shi, Xin-Wei Gong, Hai-Qing Gong, Pei-Ji Liang, Pu-Ming Zhang, Qin-Chi Lu

**Affiliations:** ^1^Department of Neurology, Ren Ji Hospital, School of Medicine, Shanghai Jiao Tong University, 1630 Dong-Fang Road, Shanghai 200127, China; ^2^School of Biomedical Engineering, Shanghai Jiao Tong University, 800 Dong-Chuan Road, Shanghai 200240, China

## Abstract

The hippocampus plays an important role in the genesis of mesial temporal lobe epilepsy, and the entorhinal cortex (EC) may affect the hippocampal network activity because of the heavy interconnection between them. However, the mechanism by which the EC affects the discharge patterns and the transmission mode of epileptiform discharges within the hippocampus needs further study. Here, multielectrode recording techniques were used to study the spatiotemporal characteristics of epileptiform discharges in adult mouse hippocampal slices and combined EC-hippocampal slices and determine whether and how the EC affects the hippocampal neuron discharge patterns. The results showed that low-Mg^2+^ artificial cerebrospinal fluid induced interictal discharges in hippocampal slices, whereas, in combined EC-hippocampal slices the discharge pattern was alternated between interictal and ictal discharges, and ictal discharges initiated in the EC and propagated to the hippocampus. The pharmacological effect of the antiepileptic drug valproate (VPA) was tested. VPA reversibly suppressed the frequency of interictal discharges but did not change the initiation site and propagation speed, and it completely blocked ictal discharges. Our results suggested that EC was necessary for the hippocampal ictal discharges, and ictal discharges were more sensitive than interictal discharges in response to VPA.

## 1. Introduction

Mesial temporal lobe epilepsy (mTLE) is the most common type of intractable epilepsy. It is closely associated with malfunctions of the mesial temporal lobe structures, such as the hippocampus and the entorhinal cortex (EC), which are heavily interconnected [1, 2]. It has been commonly accepted that the hippocampus plays an important role in the genesis of mTLE [3]. Several studies have investigated the cellular and network mechanisms of epileptiform discharges in the hippocampus [4–6]. de Curtis and Avanzini [7] and McCormick and Contreras [8] reported that the mechanisms of epileptiform discharges mainly depend on intrinsic neuronal properties, recurrent synaptic interconnections, and nonsynaptic interactions among closely located neurons, which lead to excessive neuronal synchronization. In addition to the hippocampus, several clinical cases of mTLE showed significant pathologic changes in other limbic structures, such as the EC and the amygdale [9, 10]. Additionally, observations in animal models indicated that the epileptogenic zone was broad, and the substrate for seizure generation was distributed over several limbic structures [11], including the hippocampus, EC, and amygdala.

Epileptiform activities derive from the imbalance between excitatory and inhibitory synaptic transmission in networks [8], which appear as ictal and interictal discharges. Ictal discharges (also termed seizures) typically last for a few tens of seconds to several minutes and represent the primary clinical burden of an active epileptic condition. Interictal discharges exist between seizures and usually last for a couple of hundred milliseconds to a few seconds [12]. Studies have shown that epileptiform activities observed in isolated hippocampal slices obtained from adult animals were mostly interictal discharges, which initiated from CA3a/b regions and propagated bidirectionally to CA3c and CA1 regions [13], while prolonged ictal discharges that resemble limbic seizures were rarely observed, which was consistent with our previous studies [14, 15]. On the other hand, studies using extracellular field potential recordings have confirmed that ictal discharges originated in the EC and then propagated to the hippocampus in combined EC-hippocampal slices from the adult rats [13]. Compared with the isolated hippocampal slice, the combined slice preserves the EC and its fiber connection with hippocampus. However, the mechanism by which the EC affects the discharge patterns and the transmission mode of interictal and ictal discharges within the hippocampus needs to be studied further.

In the present study, hippocampal slices and combined EC-hippocampal slices of adult male C57BL/6 mice were prepared, and low-Mg^2+^ artificial cerebrospinal fluid (ACSF) was adopted to induce epileptiform discharges in the slices, which results in epileptiform discharges by unblocking the N-methyl-D-aspartate (NMDA) receptors [6]. Microelectrode array (MEA), which has a high resolution and precision in both temporal and spatial domains, was used to record electrical signals in relevant areas simultaneously. Valproate (VPA), one of the major antiepileptic drugs (AEDs) commonly used in clinical practice, was used to examine its pharmacological effects on the different epileptiform discharges in the two slice models.

Our data showed that low-Mg^2+^ ACSF induced interictal discharges in the hippocampal slices while inducing alternating interictal and ictal discharges in the combined EC-hippocampal slices. Interictal discharges initiated in CA3a and propagated bidirectionally to CA3c and CA1 regions, while ictal discharges originated from the EC and propagated to the hippocampus. VPA reversibly suppressed the frequency of interictal discharges, while completely blocking the ictal discharges. The results suggested that EC was necessary for ictal discharges in the low-Mg^2+^-induced epileptic hippocampal slice models, and ictal discharges were more sensitive than interictal discharges in response to VPA.

## 2. Materials and Methods

### 2.1. Combined EC-Hippocampal Slice and Hippocampal Slice Preparation

Adult male C57BL/6 mice aged 12–16 weeks were purchased from the Shanghai Institutes for Biological Sciences. All animal experiments were approved by the Ethic Committee, School of Biomedical Engineering, Shanghai Jiao Tong University. All efforts were made to minimize the number of animals used and their suffering.

**Figure 1:**
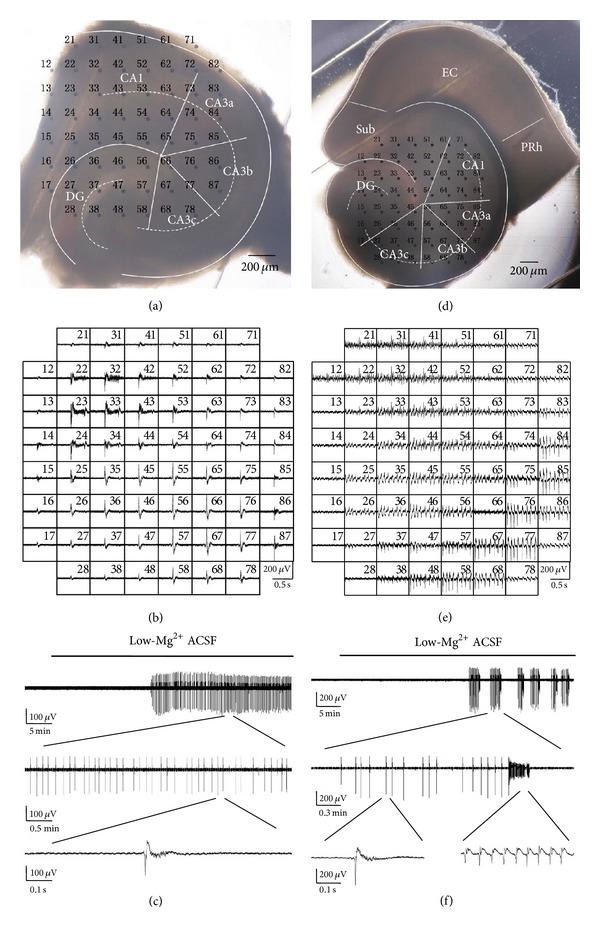
Slices mounted on MEA and low-Mg^2+^-induced epileptiform discharges. (a) An image of an example of a hippocampal slice mounted on MEA. The black dots indicate the electrodes (60 channels) of the MEA, with each electrode number labeled at its top left corner. (b) Low-Mg^2+^-induced epileptiform discharges recorded by the MEA. Each data window represented the recording from one electrode, with each electrode number labeled at its top right corner. (c) A portion of raw data recorded by one electrode (number 87), which showed epileptiform discharges in the CA3b region, with epileptiform activities presented using different time scales. (d) An image of an example of combined EC-hippocampal slice mounted on MEA. (e) Low-Mg^2+^-induced ictal discharges recorded by the MEA. (f) A portion of raw data recorded by one electrode (number 67), which showed epileptiform discharges in the CA3b region of the combined slice. There were two types of epileptiform discharges recorded in the hippocampal area: interictal and ictal discharges. Each discharge cycle consisted of several minutes of interictal discharges and tens of seconds of ictal discharge. After a resting period, this alternating interictal and ictal discharge pattern repeated.

All animals were handled and decapitated under deep isoflurane anesthesia. The brain was rapidly removed and placed in oxygenated (95% O_2_ and 5% CO_2_), ice-cold ACSF for 5 min. The composition of the normal ACSF was as follows (in mM): NaCl 124.0, KCl 3.5, CaCl_2_ 2.5, NaH_2_PO_4_ 1.2, MgCl_2_·6H_2_O 1.3, NaHCO_3_ 25.0, and glucose 10.0 (pH 7.4). The cerebellum was removed, and a sagittal cut separated the cerebral hemispheres. The cerebral hemispheres were positioned with the medial surface on a wet cold filter paper. The dorsal cortex of each hemisphere was cut parallel to the rostral/caudal axis and removed (<2 mm thick). The rostral two-thirds of the brain were removed from the caudal third, and all diencephalic and midbrain structures were subsequently removed from the caudal third. The dorsal side of the trimmed tissue was glued down to the base plate of the vibratome (Series 1000, Tissue Sectioning System, Vibratome, Natural Genetic Ltd., USA), with the caudal end towards the blade that was secured at an angle of 10°. Next, combined EC-hippocampal slices (400 *μ*m) were incubated in oxygenated ACSF at 28°C for at least 2 hours before use. In our experiments, only one slice was adopted from each mouse, with the slices obtained from similar positions in the trimmed tissue. The combined slice contained the hippocampus, dentate gyrus (DG), subiculum (Sub), EC, and part of the perirhinal cortex (PRh) (Figure 1(d)). To prepare hippocampal slices (Figure 1(a)), the EC and PRh regions were removed from the combined slices. Epileptiform activities were induced by low-Mg^2+^ ACSF, in which MgCl_2_·6H_2_O was omitted from the normal ACSF without substitution.

### 2.2. Electrophysiological Recordings

Multichannel recording system (MEA60, Multichannel Systems GmbH, Germany) was used to record electrical activities, and the microelectrode array (MEA) consists of 60 electrodes (electrode diameter is 30 *μ*m and the tip-to-tip interelectrode space is 200 *μ*m) (Figure 1(a)).

The slice was quickly transferred to the MEA recording chamber. A nylon mesh was used to immobilize the slice. The hippocampal area of the slice was placed in the recording area (Figures 1(a) and 1(d)), to record the electrical activities in the hippocampus. In other experiments, we placed the EC with parts of CA1 in the recording area (Figure 4(a)) to analyze the relationship of epileptiform discharges in the EC and the hippocampus. The slice was perfused continuously with oxygenated ACSF which was maintained at 35-36°C with a temperature control unit (Thermostat HC-X, Multichannel Systems GmbH, Germany) and at a flow rate of 2 mL/min with a peristaltic pump (Ismatec SA, USA). An Olympus microscope (Olympus, Japan) and camera (Olympus, Japan) were used to observe the relative position of the slice on the MEA and capture images. All data were recorded with a 60-channel amplifier (single-ended amplifier, bandwidth 1 Hz–3.4 kHz, amplification 1200x, amplifier input impedance >10^10^ Ω, and output impedance 330 Ω) and sampled at 20 kHz. The data were displayed on the computer screen (Figures 1(b) and 1(e)) and stored simultaneously for off-line analysis.

### 2.3. Chemicals and Drugs

VPA was purchased from Sigma-Aldrich (USA), and the other chemicals were acquired from Sinopharm Chemical Reagent Co., Ltd (SCRC, China). During the experiments, the drugs were dissolved in the low-Mg^2+^ ACSF.

### 2.4. Data Analysis

Epileptiform activities appear as interictal discharges, as well as tonic-clonic ictal discharges [8]. Interictal discharges usually terminate within a couple of hundred milliseconds to a few seconds. Ictal discharges typically last for a few tens of seconds to several minutes [12]. In the present study, the epileptiform discharges were defined as interictal if they lasted for less than 1 s (usually between 100 and 300 ms) and occurred at frequency of 0.1~0.3 Hz. Ictal discharges were defined as prolonged events which lasted for 10~30 s and occurred at a frequency of 0.003~0.006 Hz.

Off-line data analysis was performed with MC_Rack 4.1.1 (Multichannel Systems GmbH, Germany), Matlab 7.10.0 (Mathworks, USA), and SPSS Statistics 17.0 (IBM, USA). Raw data were separated into field potential (FP) and multiunit activity (MUA) by 1–100 Hz band-pass and 200 Hz high-pass filtering, respectively. MUA (data not shown) was not obvious in some slices; therefore, FP was used for further analysis. The epileptiform FP was determined when the negative-positive peak of signal exceeded four times the standard deviations from the mean value of 500-ms baseline (no obvious epileptiform discharges). The FPs of the interictal discharges in the hippocampal stratum pyramidale exhibited negative-positive waveforms [14]; thus, in the present study, the initiation and propagation of the interictal discharges were determined by comparing the timing of the first negative peaks of the FP recorded along the stratum pyramidale, and the earliest site of the negative peak of the FP was defined as the initiation site. The propagation speed of the interictal discharges was measured according to the tip-to-tip distance between the adjacent electrodes and the relative time delay of the first negative peak of the FP along the stratum pyramidale. While in the propagation route of the ictal discharges, epileptic waveforms could exhibit negative-positive (stratum pyramidale) as well as positive-negative (stratum radiatum and stratum lacunosum-moleculare) characteristics; therefore, the initiation and propagation of the ictal discharges were determined by comparing the timing of the first negative-positive peaks of the FP recorded by all electrodes in the combined slice. The data are expressed as the means ± SEM. Statistical comparisons were made using paired *t*-test or one-way ANOVA test. The level of significance was set at *P* < 0.05.

## 3. Results

### 3.1. Low-Mg^2+^-Induced Epileptiform Discharges

In our experiments, the application of low-Mg^2+^ ACSF consistently induced interictal discharges in hippocampal slices. Figures 1(a)–1(c) show an example of a hippocampal slice mounted on MEA and low-Mg^2+^-induced interictal discharges. The onset of the interictal discharges in different hippocampal slices appeared with different time delays, ranging between 10 and 20 min (16.7 ± 2.5 min, *n* = 5) after the low-Mg^2+^ ACSF perfusion began. The frequency of the interictal discharges was 0.23 ± 0.05 Hz (*n* = 5). The durations of the interictal discharges in different subregions were 184.9 ± 51.2 (CA3a), 191.0 ± 41.2 (CA3b), 176.7 ± 32.3 (CA3c), 175.0 ± 37.7 (CA1), and 151.3 ± 29.1 (DG) ms. There was no significant difference between the durations of interictal discharges in these subregions in hippocampal slices (*P* > 0.05, ANOVA, *n* = 5, Table 1).

Low-Mg^2+^ ACSF only induced interictal discharges in the isolated hippocampal slices; in that case, what was the discharge pattern in the combined slices? To compare the epileptiform discharge patterns in the isolated hippocampal slices and combined EC-hippocampal slices, the hippocampus of the combined slices was placed in the recording area. Figures 1(d)–1(f) show an example of a combined EC-hippocampal slice mounted on MEA and low-Mg^2+^-induced epileptiform discharges. The onset of the epileptiform activity in different slices was 10~25 min (17.6 ± 6.5 min, *n* = 5) after low-Mg^2+^ ACSF perfusion began. There were two types of epileptiform discharges recorded in the hippocampal area: interictal and ictal discharges. Each discharge cycle consisted of several minutes of interictal discharges (4.7 ± 2.7 min, *n* = 5) and tens of seconds of ictal discharge (14.5 ± 1.3 s, *n* = 5) (Figure 1(f)). After a resting period (1.3 ± 0.7 min, *n* = 5), this alternating interictal and ictal discharge pattern repeated. The frequency of these interictal discharges was 0.24 ± 0.04 Hz (*n* = 5), and the discharge durations in different subregions were 218.1 ± 20.6 (CA3a), 222.4 ± 16.6 (CA3b), 222.6 ± 11.3 (CA3c), 211.4 ± 63.5 (CA1), and 184.4 ± 18.8 (DG) ms. There was no significant difference between these durations (*P* > 0.05, ANOVA, *n* = 5, Table 1). The ictal discharges were usually prolonged activities, which occurred at the frequency of 0.004 ± 0.001 Hz (*n* = 5) and were characterized by rhythmic oscillations of 7.17 ± 2.75 Hz (*n* = 5). The durations of the ictal discharges in different subregions were 14.6 ± 1.7 (CA3a), 14.6 ± 1.3 (CA3b), 14.5 ± 1.5 (CA3c), 14.5 ± 1.4 (CA1), and 14.6 ± 1.3 (DG) s. There was no significant difference between these durations of ictal discharges (*P* > 0.05, ANOVA, *n* = 5, Table 1).

### 3.2. Initiation and Propagation of Interictal Discharges in Hippocampal Slices

From the above results, we found that ictal discharges were only observed in the combined slices, which was closely related to the presence of the EC. How will the EC affect the epileptiform discharge pattern in the hippocampus? In the following study, the exact propagation modes of different types of discharges within the hippocampal network in the two slice models were analyzed.

**Figure 2:**
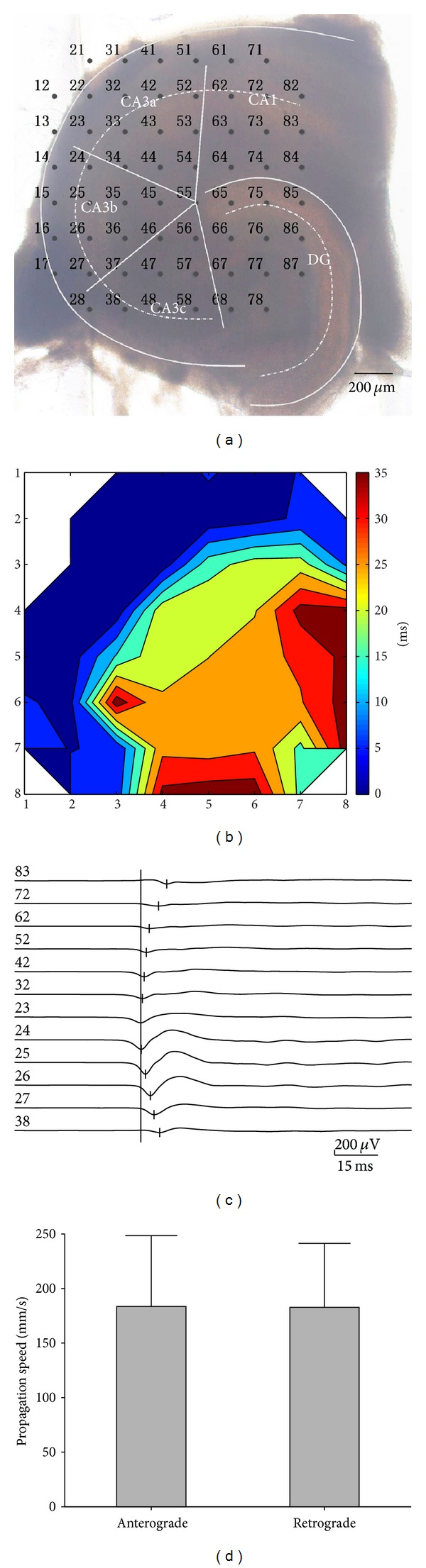
Initiation and propagation of interictal discharges in hippocampal slices. (a) An image of a hippocampal slice mounted on MEA. (b) Contour plot of averaged time delays of the FP relative to signals recorded by electrode number 23. The *X* and *Y* coordinates denote the positions of electrodes. (c) The relative time delay of first negative peak of FP recorded along the stratum pyramidale. The interictal discharges initiated from the CA3a region and propagated bidirectionally to the CA1 region (anterograde) and the CA3c region (retrograde). (d) Averaged propagation speeds of interictal discharges in hippocampal slices (*P* > 0.05, paired *t*-test, *n* = 5).

Figure 2(b) shows the contour plot of the averaged time delays of the FP relative to signals recorded by electrode number 23 on the representative hippocampal slice shown in Figure 2(a). The interictal discharge was first observed in the CA3 region and propagated to the other hippocampal regions. The time delays of the first negative peaks of FP recorded along the stratum pyramidale were compared, and the earliest site of the negative peak of FP was defined as the initiation site. As shown in Figure 2(c), the interictal discharges initiated in CA3a (electrode number 23) and propagated bidirectionally to the CA1 (anterograde) and CA3c (retrograde). The data obtained from the other 4 slices showed that the interictal discharges presented similar initiation and propagation patterns. Statistical analysis showed that the propagation speeds of the interictal discharges in the hippocampal slices were 183.5 ± 64.9 mm/s (anterograde, from CA3a to CA1) and 182.7 ± 58.7 mm/s (retrograde, from CA3a to CA3c). There was no significant difference between these two speeds (Figure 2(d), *P* > 0.05, paired *t*-test, *n* = 5).

### 3.3. Initiation and Propagation of Interictal and Ictal Discharges in Combined EC-Hippocampal Slices

**Figure 3:**
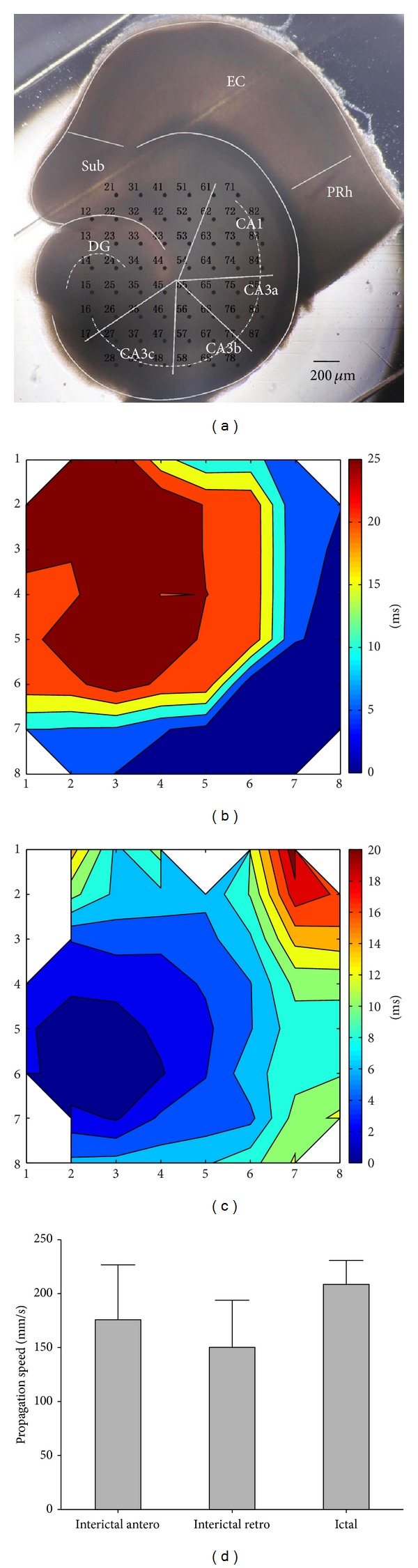
Initiation and propagation of interictal and ictal discharges in the hippocampal area of the combined EC-hippocampal slices. (a) An image of an example of a combined slice mounted on MEA. (b) Contour plot of averaged time delays of the FP relative to signals of electrode number 85. The *X* and *Y* coordinates denote the positions of electrodes. (c) Contour plot of averaged time delays of the FP relative to signals of electrode number 25. (d) Averaged propagation speeds of interictal discharges and ictal discharges in the combined slices. antero: anterograde propagation speed; retro: retrograde propagation speed (*P* > 0.05, ANOVA, *n* = 5).

The initiation and propagation of the two types of discharges in the combined EC-hippocampal slices were analyzed.  Figure 3(b) shows the contour plot of averaged time delays of the FP (interictal discharge) relative to the signals of electrode number 85 on the representative combined slice in Figure 3(a). The interictal discharges initiated in CA3a and propagated bidirectionally to the CA1 (anterograde) and CA3c (retrograde).  Figure 3(c) shows the contour plot of the averaged time delays of the FP (ictal discharge) relative to the signals of electrode number 25 on the representative combined slice in Figure 3(a). The ictal discharge was first observed in DG and propagated to CA3c, CA3b, CA3a, and CA1 along the stratum pyramidale. The data obtained from the other 4 slices showed similar discharge patterns.

From the above results, we found that the initiation site and propagation direction of the interictal and ictal discharges within the hippocampus were different. The interictal discharges initiated in CA3a and propagated bidirectionally to CA1 and CA3c, whereas the ictal discharge was first observed in the DG and propagated from the CA3c to CA1 along the stratum pyramidale. Furthermore, the propagation speeds of the interictal and ictal discharges in the hippocampal area of combined slices were analyzed. Statistical analysis showed that the propagation speeds of the interictal discharges were 175.8 ± 50.9 mm/s (anterograde, from CA3a to CA1) and 150.2 ± 43.7 mm/s (retrograde, from CA3a to CA3c), and the propagation speed of the ictal discharge was 208.6 ± 22.2 mm/s. There were no significant differences between the above three speeds (Figure 3(d), *P* > 0.05, ANOVA, *n* = 5).

### 3.4. Interactions between EC and CA1

**Figure 4:**
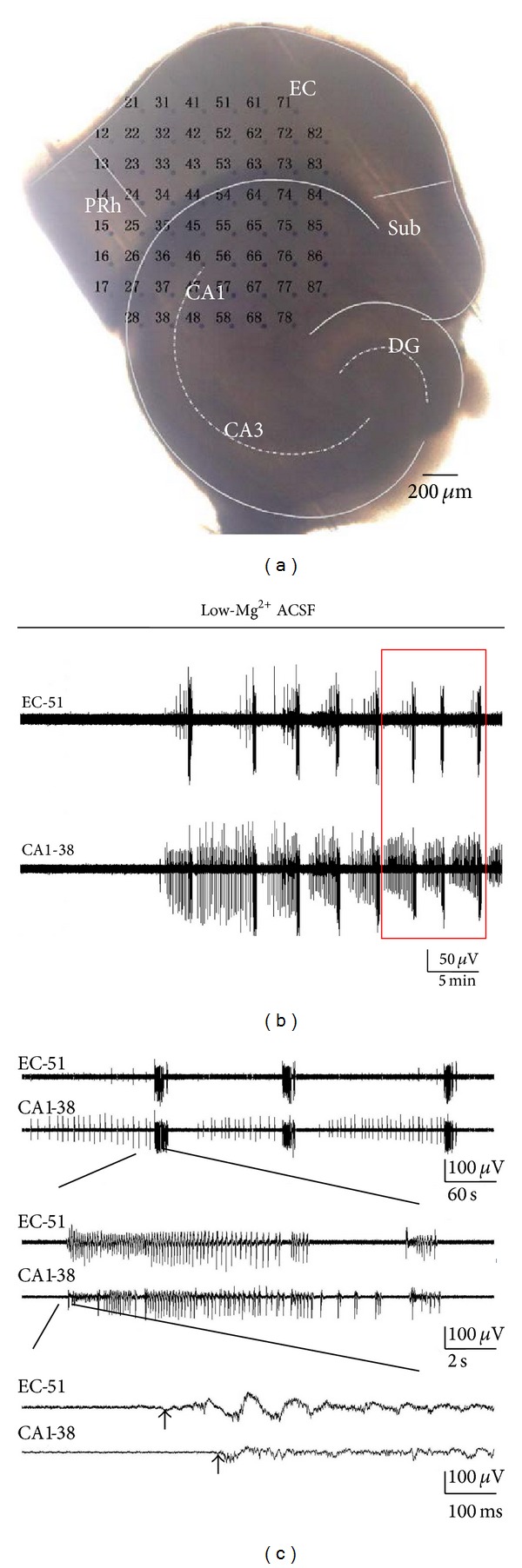
Epileptiform discharges in the EC and CA1 regions of the combined EC-hippocampal slice. (a) An example of a combined slice mounted on MEA. (b) Low-Mg^2+^-induced epileptiform discharges in the EC and CA1 regions. The FP recorded by two electrodes (numbers 51 and 38 in (a)), which presented epileptiform activities in the EC and CA1 areas. (c) Time delays of ictal discharges with expanded time scales (red box in (b)).

To explore the characteristics of epileptiform discharges in EC, as well as the relationship between the epileptiform discharges in the EC and hippocampus, the EC, along with parts of CA1, was placed in the recording area in the following experiments (Figure 4(a)).

As shown in Figure 4(b), the continuous application of low-Mg^2+^ ACSF for 14.4 ± 2.1 min (*n* = 5) resulted in epileptiform discharges in EC, and the ictal and interictal discharges were recorded. The interictal discharges in EC occurred irregularly. They generally appeared before an ictal discharge, but, in some cases, they did not appear. Therefore, we did not analyze these interictal discharges in EC in the present study. The duration of the ictal discharges in the EC was 14.4 ± 4.9 s, which occurred at frequency of 0.006 ± 0.001 Hz (*n* = 5). The ictal discharges consisted of rhythmic oscillations of 7.35 ± 0.65 Hz (*n* = 5).

In the CA1 area of combined slices, the interictal and ictal discharges were recorded with the consistent application of low-Mg^2+^ ACSF, and the epileptiform discharge pattern was consistent with that of the combined slices, the hippocampal areas of which were placed in the recording area, as mentioned in Section 3.1. Statistical analysis showed that the interictal discharges appeared with a frequency of 0.21 ± 0.05 Hz, and the duration was 199.9 ± 37.1 ms (*n* = 5). The ictal discharges in the CA1 appeared with the frequency of 0.006 ± 0.001 Hz, which was characterized by rhythmic oscillations of 7.08 ± 2.01 Hz (*n* = 5). The duration of the ictal discharges was 15.5 ± 3.7 s (*n* = 5).

**Figure 5:**
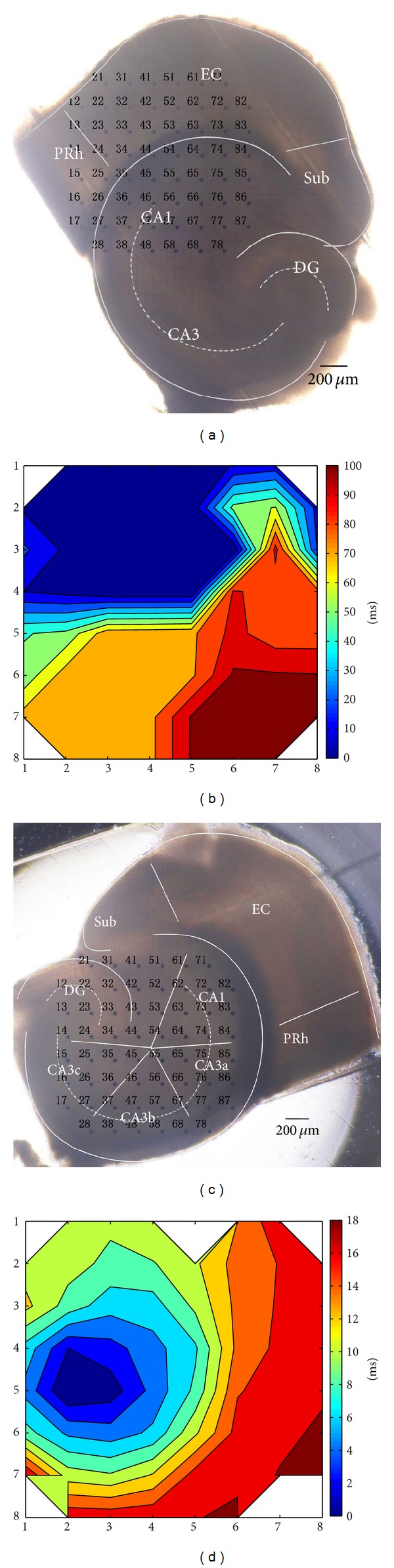
Propagation of ictal discharges in the combined EC-hippocampal slice. (a) An example of a combined slice mounted on MEA. (b) Contour plot of the ictal discharge time delays of the FP relative to signals of electrode number 51 in (a). (c) An example of a combined slice mounted on MEA. (d) Contour plot of the ictal discharge time delays of the FP relative to signals of electrode number 24 in (c).

In the combined slices, the ictal discharges occurred in both EC and CA1. As shown in Figure 5(a), both EC and CA1 regions were covered by a few electrodes. We calculated the mean onset time of the electrodes within the two regions and then analyzed the time delay between the EC and CA1 (80.4 ± 18.0 ms, *n* = 5), which inferred that the ictal discharges in the combined slices were observed earlier in EC than in CA1 (Figure 5(b)).

Then, what was the exact propagation pathway of the ictal discharges in the hippocampus? We placed the hippocampal area of the slice in the recording area, to record the electrical activities in the hippocampus (Figure 5(c)). As shown in Figure 5(d), ictal discharge was first observed in the DG and then propagated to the CA3c, CA3b, CA3a, and CA1 areas along the stratum pyramidale. Therefore, we inferred that the EC produced ictal discharges, and the ictal discharges propagated via the DG, CA3c, CA3b, and CA3a to the CA1 area in the hippocampus.

### 3.5. Effects of VPA on the Interictal Discharges in Hippocampal Slices

**Figure 6:**
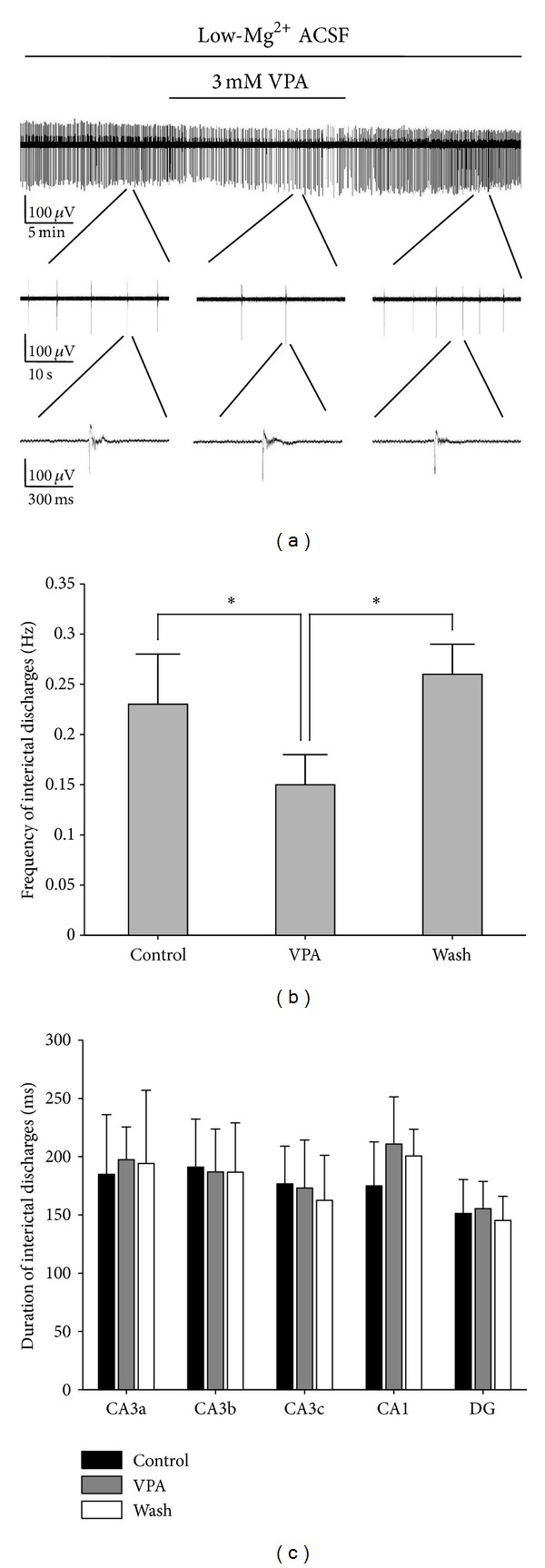
Effects of VPA on low-Mg^2+^-induced interictal discharges in hippocampal slices. (a) Long-term display of the epileptiform discharges before, during, and after 3 mM VPA application, which represented the recordings of electrode number 87 in the CA3b region (Figure 1(a)). (b) Effects of VPA on interictal discharge frequency. There were significant differences between the VPA group and the Control/Wash (*P < 0.05, ANOVA, *n* = 5). (c) Effects of VPA on interictal discharge duration. There were no significant differences between the VPA group and the Control/Wash (*P* > 0.05, ANOVA, *n* = 5).

VPA is one of the major antiepileptic drugs commonly used in clinical practice [16]. The pharmacological effects of VPA on epileptiform discharges were examined on the hippocampal slices. Application of 3 mM VPA reversibly suppressed the frequency of the interictal discharges (Figure 6(b)). The frequencies of the interictal discharges measured before, during, and after the application of VPA were 0.23 ± 0.05, 0.15 ± 0.03, and 0.26 ± 0.03 Hz, respectively. There were significant differences between the VPA group and the Control/Wash (*P < 0.05, ANOVA, *n* = 5).  Figure 6(c) shows the duration of the interictal discharges in different regions (CA3a, CA3b, CA3c, CA1, and DG) of the hippocampal slice before (Control), during (VPA), and after (Wash) the application of VPA. There were no significant differences between the duration of VPA group and the Control/Wash (*P* > 0.05, ANOVA, *n* = 5, Table 3).

**Figure 7:**
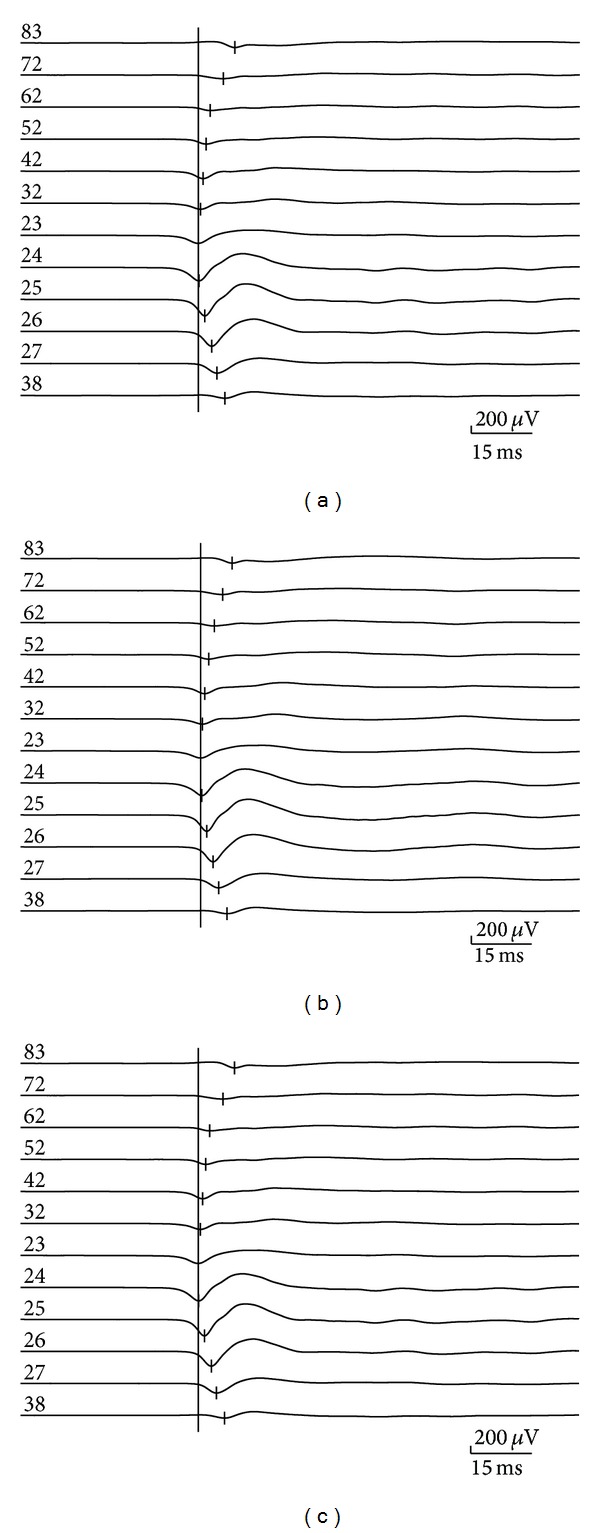
Effects of VPA on the initiation and propagation of the interictal discharges. The initiation and propagation of interictal discharge in an example of a hippocampal slice (Figure 2(a)) before (a), during (b), and after (c) application of 3 mM VPA.

We further investigated whether VPA changed the initiation and propagation of the interictal discharges in the hippocampal slices.  Figure 7 shows the relative times of the first negative peak of FP before, during, and after application of 3 mM VPA. Before dosing, the interictal discharges initiated from CA3a and propagated bidirectionally to the CA1 (anterograde) and CA3c (retrograde) (*n* = 5). VPA did not significantly change the initiation site and the propagation direction. The statistical analysis of the results obtained from 5 slices showed that the anterograde speeds (from CA3a to CA1) and retrograde speeds (from CA3a to CA3c) in the hippocampal slices were not affected significantly during the VPA perfusion (Table 2).

Therefore, the inhibitory effect of VPA was mainly reducing the interictal discharge frequency, while the discharge duration, as well as the initiation site and propagation speed, was not affected significantly.

### 3.6. Effects of VPA on the Interictal and Ictal Discharges in Combined EC-Hippocampal Slices

**Figure 8:**
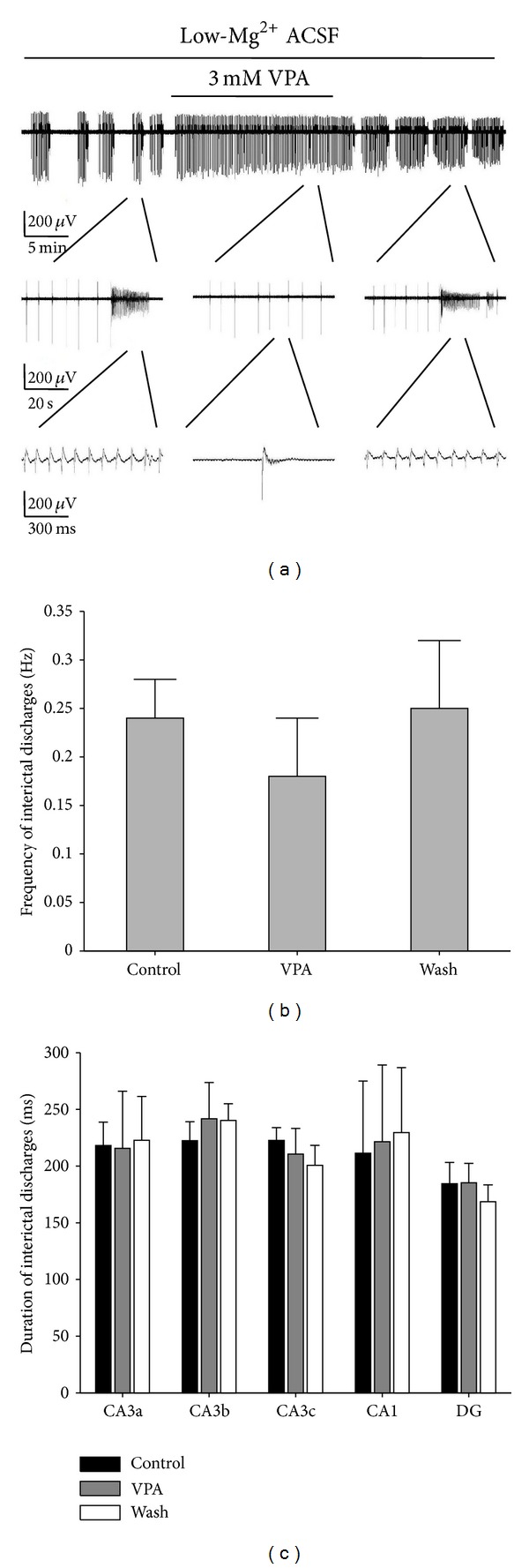
Effects of 3 mM VPA on low-Mg^2+^-induced epileptiform discharges in the hippocampal area of combined slices. (a) Long-term display of low-Mg^2+^-induced epileptiform discharges before, during, and after VPA application, which represents the recordings of electrode number 67 in the CA3b region (Figure 1(d)). (b) Effects of 3 mM VPA on interictal discharge frequency. There are no significant differences between the VPA group and the Control/Wash (*P* > 0.05, ANOVA, *n* = 5). (c) Effects of 3 mM VPA on interictal discharge duration. There are no significant differences between the VPA group and the Control/Wash (*P* > 0.05, ANOVA, *n* = 5).

Figure 8(a) shows the long-term display of low-Mg^2+^-induced epileptiform discharges before, during, and after 3 mM VPA application, which represents the recordings of one electrode (number 67) in CA3b region (Figure 1(d)) of one combined slice. The other 4 slices showed similar results. VPA reversibly changed the discharge pattern in the combined slices. The ictal discharges were completely blocked by VPA, whereas the interictal discharges were still preserved. The ictal discharges appeared again after the washout of VPA, with a frequency of 0.005 ± 0.002 Hz and consisted of a train of rhythmic oscillations of 7.72 ± 2.33 Hz (*n* = 5). The durations of the ictal discharges in different regions of the hippocampal area were 17.0 ± 7.2 (CA3a), 17.1 ± 7.3 (CA3b), 16.9 ± 7.2 (CA3c), 17.2 ± 7.3 (CA1), and 16.9 ± 7.3 (DG) s (*P* > 0.05, ANOVA, *n* = 5). The frequencies of interictal discharges measured before, during, and after application of VPA were 0.24 ± 0.04, 0.18 ± 0.06, and 0.25 ± 0.07 Hz, respectively. There were no significant differences between the VPA group and the Control/Wash (*P* > 0.05, ANOVA, *n* = 5, Figure 8(b)).  Figure 8(c) shows the duration of the interictal discharges in different regions (CA3a, CA3b, CA3c, CA1, and the DG) of the combined slice before (Control), during (VPA), and after (Wash) application of VPA. There were no significant differences between the duration of VPA group and the Control/Wash (*P* > 0.05, ANOVA, *n* = 5, Table 3).

We further investigated the effects of VPA on the initiation and propagation of the interictal and ictal discharges in the combined slices. Before dosing (Control), the interictal discharges initiated from the CA3a region and propagated bidirectionally to the CA1 (anterograde) region and the CA3c region (retrograde) (*n* = 5). VPA did not significantly change the initiation site and propagation direction. The anterograde (from CA3a to CA1) and retrograde speeds (from CA3a to CA3c) were not significantly affected during the VPA perfusion (Table 2). The ictal discharges were completely blocked by VPA. After the washout of VPA, the ictal discharges appeared again and propagated from the DG to CA3c, CA3b, CA3a, and CA1 (*n* = 5).

From the above results, we found that the inhibitory effect of VPA completely blocked the ictal discharges but had no significant effect on the interictal discharges.

### 3.7. Effects of VPA on the Epileptiform Discharges of EC/CA1

**Figure 9:**
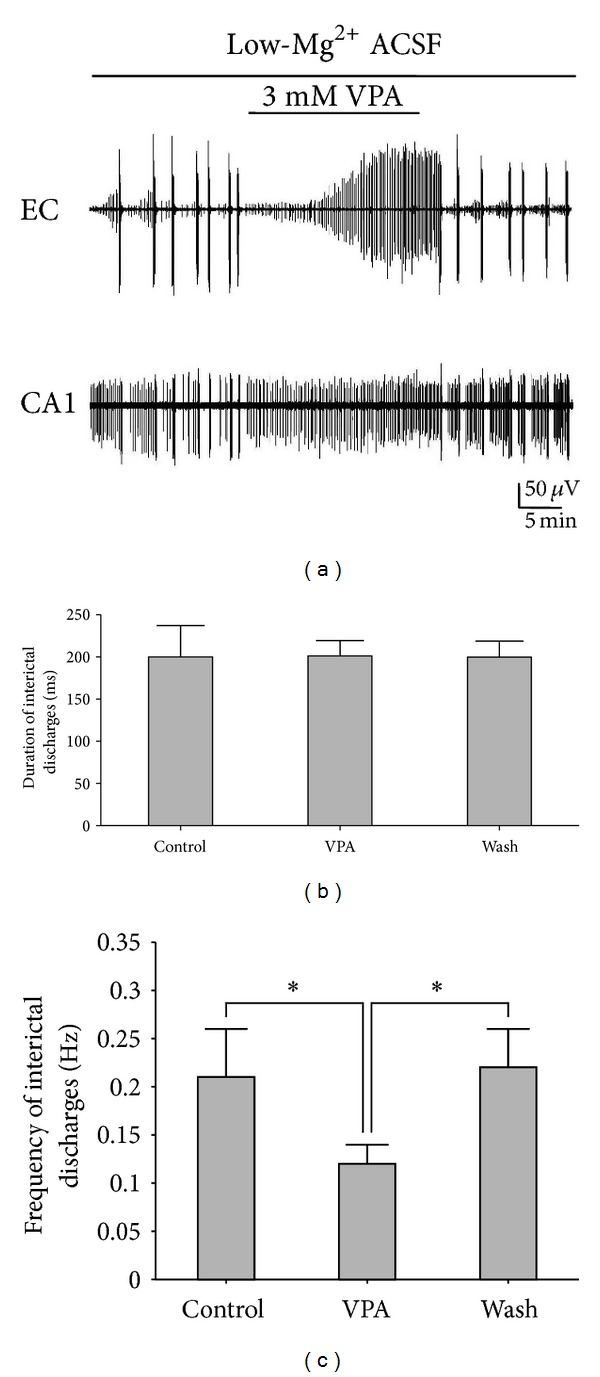
Effects of VPA on the epileptiform discharges in the EC and CA1 areas of the combined slices. (a) Long-term display of the effects of VPA (before, during, and after VPA application) on the discharge patterns in the EC and CA1 areas of an example of a combined slice. (b) The statistical analysis of VPA effects on duration of interictal discharges in CA1. There were no significant differences between the VPA group and the Control/Wash (*P* > 0.05, ANOVA, *n* = 5). (c) The statistical analysis of VPA effects on frequency of interictal discharges in the CA1. There were significant differences between the VPA group and the Control/Wash (*P < 0.05, ANOVA, *n* = 5).

As an example shown in Figure 9(a), the application of 3 mM VPA reversibly suppressed the ictal discharges in EC, and the neural activity changed into interictal discharges. In CA1, on perfusion with VPA, the ictal discharges disappeared while the interictal discharges still existed; after the washout of VPA, the ictal discharges appeared again (Figure 9(a)). The data obtained from the other 4 slices showed that the discharge pattern in the EC/CA1 presented similar characteristics when perfused with VPA.

The interictal discharges observed in the EC during the perfusion with VPA lasted for 0.18~0.71 s (0.39 ± 0.22 s, *n* = 5), with a frequency of 0.03~0.13 Hz (0.08 ± 0.04 Hz, *n* = 5).  Figure 9(b) shows the duration of the interictal discharges in the CA1 region of the combined slices before (Control), during (VPA), and after (Wash) the application of VPA. There were no significant differences between the duration of the VPA group and the Control/Wash (*P* > 0.05, ANOVA, *n* = 5). The interictal discharge frequency measured before, during, and after application of VPA was 0.21 ± 0.05, 0.12 ± 0.02, and 0.22 ± 0.04 Hz, respectively. There were significant differences between the VPA group and the Control/Wash (*P < 0.05, ANOVA, *n* = 5, Figure 9(c)).

## 4. Discussion

### 4.1. Interictal and Ictal Discharges in the Hippocampal Slice and Combined EC-Hippocampal Slice

In the present study, the application of low-Mg^2+^ ACSF consistently induced interictal discharges in the hippocampal slices and the hippocampal area of combined slices. Our results showed that the hippocampal network itself could maintain stable interictal discharges with low-Mg^2+^ ACSF perfusion, and these interictal discharges initiated from CA3a and propagated bidirectionally to CA1 and CA3c, which was consistent with other studies [17]. Additionally, in previous studies carried in our laboratory, relevant results indicated that, in the hippocampal network, interictal discharges originated from CA3a/b regions and propagated bidirectionally to the CA3c and CA1 regions, respectively [14, 15]. Some morphological data support such anterograde and retrograde propagation mechanisms. CA3 pyramidal cells send axons back to the hilar region, in which they excite mossy cells and hilar interneurons [18]. CA3 pyramidal cells also send axons to CA1 via Schaffer collaterals [19], and sectioning of the Schaffer collaterals between CA3 and CA1 abolished interictal discharges in CA1 by preventing their propagation from CA3 [20], which further confirmed that the hippocampal CA3 was sufficient to produce interictal discharges. Studies have shown that CA3 in the hippocampus is particularly prone to be pacemakers of epileptiform discharges owing to the presence of intrinsically burst-generating neurons and strong recurrent excitatory connections [21, 22].

By comparing the different discharge patterns in the hippocampal slices and the combined slices induced by low-Mg^2+^ ACSF, it is inferred that isolated hippocampal slices could only produce interictal discharges. The ictal discharges observed in the combined slices were closely related to the presence of the EC. The combined slice preparation preserved fiber connections between the hippocampus and the EC. In neuroanatomy, neurons in EC project to the DG via the perforant pathway, granule cells of the DG project to CA3 via mossy fiber projections, and pyramidal neurons in CA3 project to CA1 via Schaffer collaterals, which form the classic trisynaptic circuit [23]. A large number of in vitro studies have shown that the EC was the initiation site of ictal discharges, and they propagate from the EC to the hippocampus when the perforant path-DG route was well-preserved [13, 24]. In our experiments, we further analyzed the exact propagation pathways within the combined slices; it was not difficult to find out that ictal discharges initiated in EC, and, within the hippocampus, they first appeared in the DG and then propagated to the CA3c, CA3b, CA3a, and CA1 regions along the stratum pyramidale.

From the above description, it could be inferred that the initiation and propagation characteristics of the two types of epileptiform discharges were different. The interictal discharges initiated in the CA3a region and propagated bidirectionally to the CA1 and CA3c regions, whereas the ictal discharges initiated in the EC and propagated to DG, CA3c, CA3b, CA3a, and CA1 along the stratum pyramidale. The clinical seizure semiology is heavily influenced by the onset and spread of seizure activity, and suppressing the generation and propagation of epileptiform discharges is an effective way to control epilepsy. The role played by the EC as the generator of ictal discharges is in line with clinical evidence that dysfunction of the EC has been documented in patients with mTLE [25], and surgical removal of the EC is essential for achieving control of drug-resistant limbic seizures [26]. Therefore, a better understanding of the epileptiform discharge initiation and propagation mode may effectively guide clinical surgical planning.

### 4.2. Effects of VPA on the Interictal and Ictal Discharges

VPA is one of the major antiepileptic drugs, which is widely used in both generalized and partial epilepsies, bipolar disorders, and neuropathic pain and as a migraine prophylaxis [27]. Its antiepileptic effects are enhancing GABA-ergic inhibitory functions and reducing NMDA-ergic excitatory functions of the nervous system [16].

In the present study, we tested the inhibitory effects of VPA on different epileptiform discharges in the hippocampal slices and the combined EC-hippocampal slices. The results showed that VPA reversibly suppressed the frequency of the interictal discharges but did not change the initiation site and propagation characteristics, which initiated in the CA3a region and propagated bidirectionally to the CA1 and CA3c regions. VPA completely blocked the ictal discharges, which initiated in EC and propagated to DG, CA3c, CA3b, CA3a, and CA1 within the hippocampus. It could be inferred that ictal discharges were more sensitive than interictal discharges in response to VPA. Our results are in keeping with clinical evidence, which indicated that interictal activity was unaffected by antiepileptic drugs (AEDs) that were effective against seizures [28]. Studies have shown that the ability of AEDs to control in vitro epileptiform activities depended mainly on the characteristics of the epileptiform discharges, such as discharge duration. AEDs could abolish prolonged (>3 s) ictal discharges but failed to block shorter epileptiform events, which were reminiscent of interictal activities [29]. In the present study, ictal discharges (>10 s) were stably suppressed by 3 mM VPA, whereas interictal discharges still existed, even though the frequency was reduced. The intracellular correlate of the interictal discharge is an overt depolarization, which is called the paroxysmal depolarizing shift (PDS), and the transition from the generation of a single PDS during the interictal discharge to prolonged ictal discharge has been associated with the gradual loss of afterhyperpolarization and the progressive appearance of prolonged afterdepolarization [30]. Traub et al. have suggested that afterdepolarization is generated by NMDA receptors, which provides a prolonged depolarization of the dendrites of neuron cells, resulting in regenerative dendritic Na^+^/Ca^2+^ spikes at 10–20 Hz, which then drive the repetitive bursts of action potentials at the soma [31]. VPA can suppress the depolarization responses mediated by glutamate receptors (NMDA receptors) [32, 33]; thus, it could block the low-Mg^2+^-induced ictal discharges in our experiment.

## 5. Conclusions

In summary, our work analyzed whether the presence of the EC had an impact on the network status, as well as the resulting changes in the epileptiform discharge patterns. Low-Mg^2+^ ACSF induced interictal discharges in the adult mouse hippocampal slices and alternating interictal and ictal discharges in the combined EC-hippocampal slices. The initiation and propagation of the interictal and ictal discharges were different. The interictal discharges initiated in CA3a and propagated bidirectionally to CA1 and CA3c; the ictal discharges initiated in EC and propagated to DG, CA3c, CA3b, CA3a, and CA1 within the hippocampus. The ictal discharges were more sensitive than the interictal discharges in response to antiepileptic drug VPA.

However, in the in vitro experiments, many factors must be regarded with caution when extrapolating the mechanisms of seizures, such as epileptic models, strain of rat, age of animals, thickness of the brain slices, and the other technical issues which would affect in vitro experiments. Owing to the complexity of the generation and propagation of epileptiform discharges, the network mechanisms of hippocampal slices and combined EC-hippocampal slices need to be studied further.

## Figures and Tables

**Table 1:** Frequency and duration of low-Mg^2+^-induced epileptiform discharges in two slice models (mean ± SEM, *n* = 5).

	Frequency (Hz)	Duration (interictal: ms/Ictal: s)				
		CA3a	CA3b	CA3c	CA1	DG
HP (interictal)	0.23 ± 0.05	184.9 ± 51.2	191.0 ± 41.2	176.7 ± 32.3	175.0 ± 37.7	151.3 ± 29.1
EC-HP (interictal)	0.24 ± 0.04	218.1 ± 20.6	222.4 ± 16.6	222.6 ± 11.3	211.4 ± 63.5	184.4 ± 18.8
EC-HP (ictal)	0.004 ± 0.001	14.6 ± 1.7	14.6 ± 1.3	14.5 ± 1.5	14.5 ± 1.4	14.6 ± 1.3

HP: hippocampal slices; EC-HP: combined EC-hippocampal slices.

**Table 2:** Statistical analysis results of VPA effects on the propagation speed of epileptiform discharges in the two slice models (mean ± SEM, *n* = 5).

Speed (mm/s)	HP (interictal)		EC-HP (interictal)		EC-HP (ictal)
	Anterograde	Retrograde	Anterograde	Retrograde	
Control	183.5 ± 64.9	182.7 ± 58.7	175.8 ± 50.9	150.2 ± 43.7	208.6 ± 22.2
VPA	150.1 ± 48.3	170.1 ± 37.6	159.4 ± 54.9	168.7 ± 54.6	/
Wash	158.3 ± 54.7	174.9 ± 49.1	172.4 ± 71.6	168.9 ± 22.5	196.0 ± 25.2

HP: hippocampal slice; EC-HP: combined EC-hippocampal slice.

**Table 3:** Statistical analysis results of VPA effects on the duration of interictal discharges in the two slice models (mean ± SEM, *n* = 5).

		Duration (ms)				
		CA3a	CA3b	CA3c	CA1	DG
HP	Control	184.9 ± 51.2	191.0 ± 41.2	176.7 ± 32.3	175.0 ± 37.7	151.3 ± 29.1
	VPA	197.4 ± 28.1	186.9 ± 36.8	173.1 ± 41.2	210.9 ± 40.5	155.4 ± 23.4
	Wash	194.1 ± 62.9	186.7 ± 42.3	162.5 ± 38.7	200.6 ± 22.9	145.3 ± 20.7

EC-HP	Control	218.1 ± 20.6	222.4 ± 16.6	222.6 ± 11.3	211.4 ± 63.5	184.4 ± 18.8
	VPA	215.6 ± 50.4	241.7 ± 32.0	210.5 ± 22.7	221.5 ± 67.6	185.2 ± 17.1
	Wash	222.8 ± 38.6	240.2 ± 14.8	200.7 ± 17.6	229.5 ± 57.3	168.5 ± 14.9

HP: hippocampal slice; EC-HP: combined EC-hippocampal slice.
